# Induction of protein aggregation and starvation response by tRNA modification defects

**DOI:** 10.1007/s00294-020-01103-w

**Published:** 2020-08-29

**Authors:** Roland Klassen, Alexander Bruch, Raffael Schaffrath

**Affiliations:** Institut für Biologie, Fachgebiet Mikrobiologie, Universität Kassel, Heinrich-Plett-Str. 40, 34132 Kassel, Germany

**Keywords:** tRNA modification, Protein aggregation, Decoding, Starvation response

## Abstract

Posttranscriptional modifications of anticodon loops contribute to the decoding efficiency of tRNAs by supporting codon recognition and loop stability. Consistently, strong synthetic growth defects are observed in yeast strains simultaneously lacking distinct anticodon loop modifications. These phenotypes are accompanied by translational inefficiency of certain mRNAs and disturbed protein homeostasis resulting in accumulation of protein aggregates. Different combinations of anticodon loop modification defects were shown to affect distinct tRNAs but provoke common transcriptional changes that are reminiscent of the cellular response to nutrient starvation. Multiple mechanisms may be involved in mediating inadequate starvation response upon loss of critical tRNA modifications. Recent evidence suggests protein aggregate induction to represent one such trigger.

## Background

During decoding of mRNA, codons are recognized by the tRNA anticodon. For efficient decoding, the tRNA must be correctly folded into an L-shaped structure and the anticodon presented in an unpaired open loop. Posttranscriptional modifications in the anticodon loop are thought to improve codon recognition and contribute to anticodon loop stability by promoting base stacking interactions, reducing the flexibility of the sugar phosphate backbone and preventing unwanted across-the-loop base pairing (Agris 2008; Sokołowski et al. 2017; Väre et al. 2017; Vendeix et al. 2012). For example, tRNA^Lys^_UUU_ contains mcm^5^s^2^U_34_ (5-methoxycarbonylmethyl-2-thiouridine at position 34) and ct^6^A_37_ (cyclic *N*^6^-threonylcarbamoyladenosine at position 37) modifications which each fulfill one or more of these tasks (Johansson et al. 2018; Miyauchi et al. 2013; Schaffrath and Leidel 2017; Thiaville et al. 2014). Both, mcm^5^s^2^U and ct^6^A are formed by multiple biosynthetic enzymes and steps. Completion of mcm^5^s^2^U synthesis is abolished at distinct steps in *elp3* and *urm1* mutants, while ct^6^A formation from the t^6^A (*N*^6^-threonylcarbamoyladenosine) precursor requires *TCD1* (Huang et al. 2005; Leidel et al. 2009; Miyauchi et al. 2013). Hence, in *elp3*, *urm1* and *tcd1* mutants, distinct pathway intermediates are formed at the target nucleosides U_34_ and A_37_. Consistent with functional redundancy, joint abrogation of mcm^5^s^2^U synthesis at different steps and prevention of t^6^A to ct^6^A conversion results in a functional defect of tRNA^Lys^_UUU_ normally carrying these modifications (Klassen et al. 2016). A similar functional redundancy exists in the tRNA^Gln^_UUG_ anticodon loop which naturally carries mcm^5^s^2^U and Ψ_38_ (pseudouridine at position 38) (Han et al. 2015; Klassen et al. 2016). Combined absence of mcm^5^s^2^U and Ψ_38_ in *elp3 deg1* or *urm1 deg1* double mutants causes a severe functional impairment of this tRNA. When formation of mcm^5^s^2^U is completely abolished by combining *elp3* and *urm1* or *elp6* and *ncs2* modifications, both, tRNA^Gln^_UUG_ and tRNA^Lys^_UUU_ are functionally impaired (Björk et al. 2007; Klassen et al. 2015; Nedialkova and Leidel 2015; Xu et al. 2019).

## Effects of modification loss on decoding and protein homeostasis

**Fig. 1:**
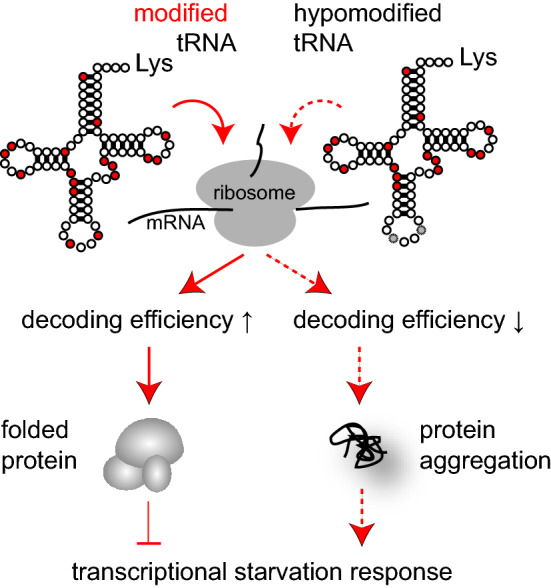
Model for the induction of a transcriptional starvation response in combined absence of anticodon loop modifications. tRNA^Lys^_UUU_ is depicted with modified positions (indicated in red). In combined *elp3 tcd1* or *urm1 tcd1* mutants, anticodon loop modifications mcm^5^s^2^U and ct^6^A are missing (indicated in grey), causing decreased decoding efficiency of cognate AAA (Lys) codons. Multiple mechanisms are discussed how such decoding defect may cause accumulation of cellular protein aggregates. New results suggest that protein aggregates are involved in triggering a subsequent transcriptional response reminiscent of nutrient starvation

In the mutants carrying combinations of tRNA modification defects, negative phenotypes and translational incompetence are routinely suppressed by overexpression of the functionally impaired tRNAs (Björk et al. 2007; Han et al. 2015; Klassen et al. 2015, 2016; Nedialkova and Leidel 2015). Elevated abundance of the hypomodified tRNA is thought to counteract the translational deficiency, which may result from increased rejection rate during the codon recognition process (Ranjan and Rodnina 2017; Rezgui et al. 2013). Another cellular consequence of such specific tRNA defects is a severe protein homeostasis disturbance, resulting in the accumulation of protein aggregates (Fig. 1) (Nedialkova and Leidel 2015). The exact mechanism how combined tRNA modification defects trigger protein aggregation is not known, but it can be assumed that ribosomal pausing is an important factor for this effect. Ribosomal pausing at CAA (Gln) and AAA (Lys) codons has been indeed demonstrated for yeast strains lacking mcm^5^s^2^U (Nedialkova and Leidel 2015), and mcm^5^s^2^U deficiency in combination with either loss of ct^6^A or Ψ_38_ likely aggravates pausing at CAA or AAA codons, respectively (Bruch et al. 2020; Klassen et al. 2016; Pollo-Oliveira et al. 2020). Such pause during translational elongation may as well increase the occurrence of ribosomal errors including + 1 frameshifts and, potentially, mistranslation due to misreading by near- or non-cognate tRNAs. An increase in + 1 frameshift rates has in fact been detected in yeast strains lacking mcm^5^s^2^U alone and in combination with ct^6^A defects (Klassen et al. 2017; Pollo-Oliveira et al. 2020; Tükenmez et al. 2015). While in such mutants, the efficiency of near/non-cognate tRNA misincorporation at the AAA or CAA codons has not been investigated, a similar effect was observed for misreading of CGC (Arg) codons by tRNA^His^_GUG_ (Khonsari and Klassen 2020). Here, absence of Pus1 dependent Ψ in the normal CGC decoder tRNA^Arg^_ICG_ increased misreading by tRNA^His^_GUG_, which is not naturally modified by Pus1. Thus, in general, impairment of a cognate tRNA upon loss of critical modifications may increase near cognate misreading by a competitor tRNA that does not rely on the same modification. In addition to effects potentially associated with ribosomal pausing, the ability of the hypomodified tRNA itself to engage in misreading might also be affected by loss of critical anticodon loop modifications. Some specific errors indeed increased in the absence of mcm^5^s^2^U (Joshi et al. 2018). However, when misreading of near cognate codons with wobble base mismatches to tRNA^Lys^_UUU_ was studied, mcm^5^s^2^U promoted rather than inhibited these types of errors (Joshi et al. 2018).

An alternative mechanism how protein aggregation might be linked to tRNA modification defects causing ribosomal pausing lies with disturbance of co-translational protein folding (Nedialkova and Leidel 2015). Support for this assumption stems from the observation of similarities in protein aggregate induction in a mcm^5^s^2^U-deficient yeast strain and a mutant lacking the ribosome-associated chaperones Ssb1/2, which are important for co-translational protein folding (Nedialkova and Leidel 2015). Thus, multiple mechanisms might link tRNA modification defects to the production of faulty proteins, which may be relevant for the common observation of impaired protein homeostasis in different tRNA modification mutants (Klassen et al. 2016; Nedialkova and Leidel 2015; Pollo-Oliveira et al. 2020; Thiaville et al. 2016; Xu et al. 2019).

## Starvation responses of tRNA modification mutants

Interestingly, different tRNA modification defects also evoke major transcriptome changes and part of these are reminiscent of the transcriptomic response to nutrient depletion. Several yeast tRNA modification mutants including those lacking mcm^5^s^2^U and ct^6^A induce *GCN4*-dependent amino acid biosynthesis genes despite the presence of amino acids in the medium (Daugeron et al. 2011; Zinshteyn and Gilbert 2013). In absence of either mcm^5^s^2^U or ct^6^A, *GCN4* induction occurred independent of the Gcn2 kinase which is activated upon binding of uncharged tRNA (Daugeron et al. 2011; Zinshteyn and Gilbert 2013). The *GCN2*-independent *GCN4* induction in these mutants suggested a non-canonical mechanism is involved in expression of general amino acid control (GAAC) genes in different tRNA modification mutants (Daugeron et al. 2011; Zinshteyn and Gilbert 2013). The recent characterization of transcriptomic changes after combined loss of mcm^5^s^2^U and either ct^6^A or Ψ_38/39_ revealed additional facets of a common starvation in response to loss of different tRNA modifications (Bruch et al. 2020).

In these strains, when grown to early exponential phase, premature transcriptional activation of genes occurred that are normally expressed only upon entry into stationary phase or nutrient depletion. This includes a loss of glucose repression and induction of nitrogen catabolite-repressed (NCR) genes in addition to the activation of different amino acid biosynthesis genes (Bruch et al. 2020). Also, autophagy (another cellular starvation response) was induced as judged from studying loss of Atg13 phosphorylation and degradation of a GFP-Atg8 fusion protein. Since NCR and autophagy are controlled by the TORC1 complex in budding yeast, these cellular responses to combined tRNA modification defects might be caused by loss or suppression of TORC1 activity (Bruch et al. 2020). Additional evidence for a role of Elp3-dependent tRNA modification in reciprocal regulation of TORC1 and TORC2 activities was obtained in a recent fission yeast study (Candiracci et al. 2019). In budding yeast, TORC1 activity also appears to be influenced by the level of uncharged tRNAs (Kamada 2017). These results suggest that the TOR complex, which represents a master regulator of growth and metabolism (Loewith and Hall 2011) might monitor the modification and charging status of tRNA. Loss of mcm^5^U or s^2^U modifications not only influences nutrient sensitive gene expression signatures, but also results in robust changes in cellular metabolism, and some of these are again reminiscent of cellular responses to nutrient starvation (Gupta et al. 2019; Karlsborn et al. 2016). Thus, apart from tRNA aminoacylation, multiple lines of evidence support an emerging role for tRNA anticodon loop modifications in the cellular signaling of nutrient availability.

## Potential mediators of nutrient signaling defects in tRNA modification mutants

Several tRNA modification defects in yeast are known to trigger *GCN4* expression in the absence of amino acid starvation. This includes not only the mcm^5^s^2^U and ct^6^A defective mutants described above, but was also observed in *deg1, pus7, rit1, trm1, trm7, mod5* and *tyw3* mutants lacking various other tRNA modifications (Chou et al. 2017; Han et al. 2018). While such amino acid starvation response appeared to be independent of the Gcn2 kinase responding to uncharged tRNA in mcm^5^s^2^U and ct^6^A defective strains, it was shown to be Gcn2 dependent in *trm7* mutants (Daugeron et al. 2011; Han et al. 2018; Zinshteyn and Gilbert 2013). In these mutants, which lack 2′-*O*-methylation of C32 and G34 in tRNA^Phe^, reduced charging of the hypomodified tRNA was observed (Han et al. 2018). Hence, the GAAC starvation response in tRNA modification mutants can be triggered in some cases by effects on the tRNA aminoacylation efficiency.

In s^2^U-deficient strains, robust metabolic changes involve increased storage carbohydrate synthesis, which normally occurs after glucose depletion (Gupta et al. 2019). Interestingly, these effects were linked to a disturbance of phosphate homeostasis. Increased trehalose synthesis likely occurs to counteract reduced intracellular phosphate levels since trehalose generation from trehalose phosphate can replenish intracellular phosphate levels. The phosphate shortage in s^2^U-deficient mutants is thought to be triggered by transcriptional and translational downregulation of *PHO* genes involved in phosphate uptake (Gupta et al. 2019). A similar mechanism might be involved in starvation like responses in other tRNA modification mutants, including those required for formation of mcm^5^U and ct^6^A, since transcriptional downregulation of *PHO* genes was observed (Chou et al. 2017). In the s^2^U-deficient strain, however, no robust transcriptional starvation response was triggered (Gupta et al. 2019), which is in contrast to the changes seen in combined mutants. Since the combined mutants exhibit growth defects exceeding those of the s^2^U-deficient strain, more robust changes might occur also at the metabolic level (Bruch et al. 2020; Klassen et al. 2016). It remains unknown, however, how exactly the transcriptional response is mediated.

Intriguingly, when studying the transcriptional induction of nutrient responsive genes in combined tRNA modification mutants, their expression was dampened upon overexpression of the very same tRNAs that conferred a suppression of growth defects (Bruch et al. 2020; Klassen et al. 2016). As outlined above, the overexpressed tRNA presumably directly counteracts the inefficiency in decoding. At the same time, the propensity to accumulate protein aggregates (see above) is significantly lowered by the tRNA overexpression constructs. Hence, protein aggregates are linked to the decoding defect and are potentially involved in the observed gene expression changes (Fig. 1). Further support for this hypothesis was obtained from studying a mutant (*zuo1*) accumulating protein aggregates independent of a tRNA modification defect (Bruch et al. 2020). In *zuo1* mutants, the ribosome-associated chaperone system is severely compromised, leading to accumulation of protein aggregates (Bruch et al. 2020). At the same time, marker genes that are subject to glucose repression or NCR become transcriptionally induced despite the presence of glucose and ammonia in the medium. Thus, protein aggregates might be mechanistically involved in mediating transcriptional changes in response to combined loss of tRNA modifications. Possibly, the proteasome-mediated turnover of normally short-lived transcription factors is altered upon cellular accumulation of protein aggregates, ultimately leading to the observed changes in gene expression signatures. Further work will be required to test this hypothesis and other potentially involved mechanisms.
